# Health of Louisville—The Weather

**Published:** 1853-07

**Authors:** 


### THE WESTERN JOURNAL

THIRD SERIES.—VOL. XII.—NO. I.

LOUISVILLE, JULY, 1853.

---

HEALTH OF LOUISVILLE—THE WEATHER.

Every physician recognises the connection between health and the weather. We expect certain diseases in a particular condition of the atmosphere, and when the condition changes we look for complaints of a different character. Extremes of temperature are esteemed unfavorable to health. Old and infirm persons are said to be more likely to die in mid-winter or mid-summer. A variable climate, where the range of the thermometer is great, is supposed to be peculiarly trying to the human constitution. Meteorology, from this intimate dependence of health upon the weather, has always been a favorite study with physicians.

We noted in our last number an extraordinary drought and elevation of temperature for our latitude and the month of June. On the 18th of May, Louisville was visited by a severe thunder shower, in which a negro man and his team were killed by lightning on Walnut street. From that time until the 3d of July, or more than six weeks, the rain that fell did not amount to three-fourths of an inch—it was only sufficient to lay the dust, two or three times, in the streets. The heat was at the same time excessive. From a meteorological table, kept by our friend, Lawrence Young, Esq., nine miles from the city, we observe that the mean temperature of the month of June, at his farm, was 75½°, and that the mercury in Fahrenheit's thermometer ranged for many days from 90° to 97°. The minimum at two o'clock any day during the month was 75°. In the city the heat was still more excessive. For the first time, we believe, in the memory of the oldest inhabitant, the thermometer indicated a temperature above blood heat—within one degree of a hundred. During several successive days it rose, in a fair exposure, above 95°.

It is interesting to remark, that during this month of tropical fever the health of the city was most excellent, and that while men pursued every branch of labor under this torrid sun with their accustomed activity, no accidents from heat—no *coups de soleil*—were reported. Bowel affections were uncommonly rare. We saw a few cases of cholera infantum, and heard of a few more in the practice of our friends, but the complaint assumed nothing of an epidemic form. Dysentery, cholera morbus, fevers, intermittent or remittent, had no existence. There have been some cases of measles and some of erysipelas, and here and there a case of typhoid fever has occurred. Of those who have suffered with this fever—always a rare complaint in Louisville—we have to record the melancholy case of a promising student of medicine, Mr. Angelo W. North, who fell a victim to the disease on the 2nd of this month.

